# Ethnic Differences in Magnesium Intake in U.S. Older Adults: Findings from NHANES 2005–2016

**DOI:** 10.3390/nu10121901

**Published:** 2018-12-04

**Authors:** Sarah E. Jackson, Lee Smith, Igor Grabovac, Sandra Haider, Jacopo Demurtas, Guillermo F. López-Sánchez, Pinar Soysal, Sarah Redsell, Ahmet Turan Isik, Lin Yang

**Affiliations:** 1 Department of Behavioural Science and Health, UCL, London WC1E 6BT, UK; 2 The Cambridge Centre for Sport and Exercise Sciences, Anglia Ruskin University, Cambridge CB1 1PT, UK; lee.smith@anglia.ac.uk; 3 Department of Social and Preventive Medicine, Centre for Public Health, Medical University Vienna, 1090 Vienna, Austria; igor.grabovac@meduniwien.ac.at (I.G.); sandra.a.haider@meduniwien.ac.at (S.H.); 4 Primary Care Department Azienda USL Toscana Sud Est, 58100 Grosseto, Italy; eritox7@gmail.com; 5 Faculty of Sports Sciences, University of Murcia, 30071 Murcia, Spain; gfls@um.es; 6 Department of Geriatric Medicine, Faculty of Medicine, Bezmialem Vakif University, 34093 Istanbul, Turkey; dr.pinarsoysal@hotmail.com; 7 Faculty of Health, Education, Medicine and Social Care, Anglia Ruskin University, Cambridge CB1 1PT, UK; sarah.redsell@anglia.ac.uk; 8 Unit for Aging Brain and Dementia, Department of Geriatric Medicine, Faculty of Medicine, Dokuz Eylul University, 35220 Izmir, Turkey; atisik@yahoo.com; 9 Department of Epidemiology, Centre for Public Health, Medical University of Vienna, 1090 Vienna, Austria; lin.yang@muv.ac.at

**Keywords:** magnesium, disparities, older adults, ethnicity, NHANES

## Abstract

Magnesium plays a crucial role in hundreds of bodily processes relevant to aging, but consumption of dietary magnesium intake has been shown to be inadequate in a large proportion of older adults. Identifying groups at risk of low magnesium intake is important for informing targeted advice. Using data from the National Health and Nutrition Examination Survey (NHANES) 2005–2016, we examined the association between ethnicity (Caucasian/African American/Hispanic/other) and magnesium intake in a large representative sample of U.S. older adults (≥65 y, *n* = 5682, mean (SD) 72.9 (0.10) y). Analyses adjusted for total energy intake and a range of relevant covariates. Overall, 83.3% of participants were not meeting the recommended level of dietary magnesium intake, ranging from 78.1% of other ethnic groups to 90.6% of African Americans. In the fully adjusted model, magnesium intake was lower among African American older adults (−13.0 mg/d, 95% CI: −18.8 to −7.2), and higher among Hispanics (14.0 mg/d, 95% CI: 7.5 to 20.5) and those from other ethnic groups (17.2, 95% CI: 3.8 to 30.5) compared with Caucasian older adults. These results highlight the need for targeted interventions to increase magnesium intake in U.S. older adults, with a focus on African Americans, in order to reduce the burden of morbidity and ethnic inequalities in health in later life.

## 1. Introduction

Magnesium is the fourth most abundant mineral in the human body and plays a pivotal role in many of its functions, including muscle function, energy metabolism, and cellular aging [1,2,3]. Accumulating evidence has demonstrated associations between inadequate magnesium intake and increased risk of a host of adverse age-associated health outcomes, including cognitive decline [4], decreased muscle performance [5], frailty [6], osteoporosis and fractures [7], diabetes [8], hypertension [9], and certain cancers [10]. Adequate magnesium intake is therefore critically important for health and physical function in later life [5,11]. Given that magnesium deficiency can easily be reversed with dietary modification or supplementation [12], identifying groups at risk of low magnesium intake is important for informing targeted advice.

A large proportion of older adults fail to meet the recommended daily allowance (RDA) for magnesium [13,14]. In the U.S., around half (48%) of people consume less than the recommended amount of magnesium from food [11], and average intake decreases with age [15]. There is some evidence that people from certain ethnic groups, such as African Americans, have particularly low intakes [15,16]. For example, in the National Health and Nutrition Examination Survey (NHANES) 1999–2000, the mean intake of dietary magnesium was significantly lower among African Americans (*n* = 810) than Caucasians (*n* = 1,913) and Hispanics (*n* = 1144) [15]. However, no studies to our knowledge have specifically examined ethnic differences in magnesium intake among older adults. In addition, the most recent available data are from almost two decades ago, since which time evidence of population-level change in magnesium intake and changes in ethnic differences in overall dietary quality have been reported. In NHANES, the prevalence of not meeting the RDA for magnesium fell by 14% between 2001/02 and 2005/06 [11]. In 2003/04, overall dietary quality was better among Hispanics than African Americans or Caucasians, with no significant difference observed between the latter two groups [17]; however, in analyses of aggregated data from 2005–2012, African Americans were found to have lower-quality diets than Caucasians and Hispanics, with no difference observed between Caucasians and Hispanics [18]. These changes limit the generalizability of existing data on ethnic differences in magnesium intake.

This study therefore aimed to examine the association between ethnicity and magnesium intake in a large, representative sample of U.S. older adults (≥65 years). Specifically, we tested for differences in magnesium intake between Caucasians, African Americans, Hispanics, and other ethnicities, adjusting for total energy intake and a range of relevant covariates.

## 2. Materials and Methods

### 2.1. Study Population

Data were from NHANES, a repeat cross-sectional survey of representative samples of the civilian non-institutionalized U.S. population, designed to provide estimates of health and nutrition status [19]. We aggregated data from older (≥65 years) respondents to the survey in six cycles conducted between 2005/06 and 2015/16. We restricted our analytic sample to respondents with complete data on dietary magnesium intake based on two 24-h dietary recalls. The NHANES obtained approval from the National Center for Health Statistics Research Ethics Review Board and participants provided written consent.

### 2.2. Dietary Assessment

A key component of the NHANES study is dietary assessment. In brief, NHANES participants were asked for two 24-h recalls of dietary intake using the USDA’s Automated Multiple-Pass method [20]. The first recall was conducted in person with a trained dietary interviewer using a standardized protocol during the physical examination (conducted in a mobile examination center). The second recall was conducted by telephone 3–10 days after the first recall. Nutrient intakes were calculated based on food intake using a revised nutrient database that converts intake of different foods recorded in each dietary recall to daily nutrient consumption for each individual.

Magnesium intake was calculated using the average of the two 24-h recalls, with implausibly high intakes excluded by removing observations above the 99th percentile. We further dichotomized the magnesium intake variable according to whether the respondent was meeting the RDA for magnesium (yes/no), using 420 mg for men and 320 mg for women [14].

Similarly, total energy intake was derived from the average of the two 24-h recalls, with observations above the 99th percentile removed.

### 2.3. Ethnicity

Ethnicity was self-reported and categorized as Caucasian, African American, Hispanic, or other.

### 2.4. Covariates

Weight and height were measured either during the physical examination in a mobile examination center or in the participant’s home as part of the NHANES data collection procedure, following standard procedures. Body mass index (BMI) was calculated as weight in kg/(height in meters)^2^. The standard definition for overweight and obesity classification [21] was used to categorize BMI: underweight (<18.5 kg/m^2^), normal weight (18.5–24.9 kg/m^2^), overweight (25.0–29.9 kg/m^2^), and obesity (≥30 kg/m^2^). For analytic purposes, we excluded those who were underweight due to potential underlying health conditions.

Self-reported sociodemographic characteristics included age, sex, education (below high school, high school, and above high school), and annual household income ($25,000 or less, $25,000 to $74,999, and $75,000 or above). Lifestyle characteristics included leisure-time physical activity and smoking status. Participants reported the number of days and minutes spent in moderate and vigorous recreational physical activities in a typical week. We summarized the total number of minutes for both activities and classified participants as physically inactive (zero moderate-to-vigorous physical activity) or active (any moderate-to-vigorous physical activity). Smoking status was classified into: never smokers (has smoked less than 100 cigarettes in their lifetime and does not smoke now), former smokers (smoked ≥100 cigarettes in their lifetime but does not smoke now), and current smokers (smoked ≥100 cigarettes in their lifetime and smokes now). Information on chronic conditions that were considered suspected correlates were extracted, including self-reported doctor-diagnosed diabetes mellitus, cardiovascular disease, arthritis, and cancer (any vs none of these).

### 2.5. Statistical Analyses

All statistical analyses were performed using STATA version 14.0 (STATA Corp., College Station, TX, USA). Survey analysis procedures were used to account for the sample weights, stratification, and clustering of the complex sampling design to ensure nationally representative estimates [22]. Sample characteristics were compared between ethnicities using linear regression for continuous variables and chi-squared tests for categorical variables. We then constructed three linear regression models to evaluate ethnic disparities in dietary magnesium intake. The first two models estimated (i) the univariate and (ii) the age-adjusted beta-coefficient of magnesium intake as a function of ethnicity, using Caucasians as the reference group. The third was a multivariable-adjusted model that included sociodemographic factors (age, sex, weight status, education level, annual household income), lifestyle factors (leisure-time physical activity, smoking status), and chronic conditions. Again, Caucasians were the reference group. All models were additionally adjusted for total energy intake, in order to estimate energy intake adjusted magnesium intake. Statistical significance was set at *p* < 0.05.

## 3. Results

**Figure 1:**
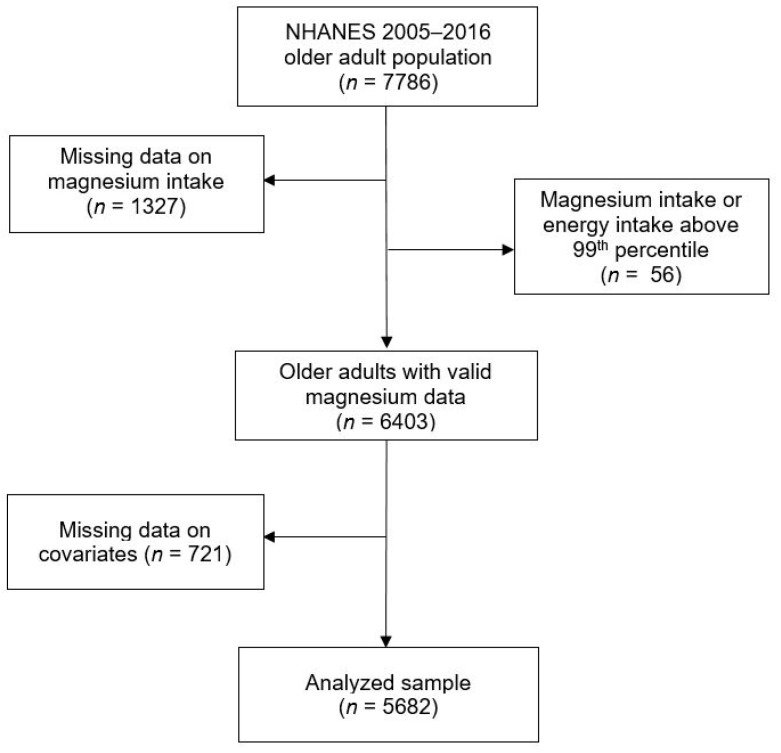
Participants flow chart for U.S. older adults (≥65 years) from the National Health and Nutrition Examination Survey (2005–2016).

Overall, 5682 adults aged ≥65 years in the six NHANES cycles with complete information on magnesium intake were included in the analysis (see Figure 1 for a flow chart of participants through the eligibility process). Participants’ mean age was 72.9 (SE 0.10) years at the time of examination, and their mean BMI was 29.0 kg/m^2^. We observed statistically significant ethnic differences in most sample characteristics, with the exception of chronic conditions (Table 1). Compared with Caucasians, a higher proportion of African American and Hispanic older adults had obesity, and more had low levels of education and low household income, more were physically inactive, and more were current smokers (Table 1). “Other” ethnic groups had the lowest prevalence of obesity and inactivity, and comparable levels of education and household income to Caucasians.

Overall, 83.3% of participants were not meeting the recommended level of dietary magnesium intake, ranging from 78.1% of other ethnic groups to 90.6% of African Americans. Table 2 summarizes univariate, age-adjusted, and multivariable-adjusted associations between ethnicity and dietary magnesium intake in linear regression models. In the univariate model, energy intake adjusted magnesium intake was significantly lower among African Americans (−19.9 mg/d, 95% CI: −26.1 to −13.7) and higher among other ethnic groups (20.7 mg/d, 95% CI: 6.7 to 34.8) compared to Caucasian older adults, but there was no significant difference in magnesium intake between Hispanics and Caucasians. The results remained similar with age adjustment. In the multivariable model, which adjusted for age, sex, weight status, education, household income, physical activity, smoking status, and chronic conditions, energy-intake-adjusted magnesium intake remained lower among African American older adults (−13.0 mg/d, 95% CI: −18.8 to −7.2), and higher among those from other ethnic groups (17.2, 95% CI: 3.8 to 30.5) compared with Caucasian older adults. In addition, a higher intake of magnesium was observed among Hispanic older adults (14.0 mg/d, 95% CI: 7.5 to 20.5) relative to Caucasians. Sensitivity analyses, in which models were stratified by sex, showed no notable differences in the pattern of results between men and women (data not shown). 

## 4. Discussion

In a large representative sample of older U.S. adults, we found that (i) the majority of U.S. older adults do not meet the RDA for magnesium intake, and (ii) ethnic differences in magnesium intake exist. After adjustment for covariates, magnesium intake was significantly lower in African Americans compared with Caucasians, and significantly higher in Hispanics and people from other ethnic groups.

The finding that the majority of U.S. older adults do not meet the RDA for magnesium is of concern given the numerous health complications associated with inadequate magnesium intake [4,5,6,7,8,9,10]. This is particularly problematic as these health complications, including cognitive decline, decreased muscle performance, frailty, osteoporosis and fractures, diabetes, hypertension, and certain cancers, are more likely to afflict older adults [4,5,6,7,8,9,10]. Therefore, promoting magnesium intake among older U.S. adults may be an effective strategy to aid in the prevention of certain conditions. In support, meta-analyses of randomized trials have shown that magnesium supplementation is associated with a small overall reduction in blood pressure [23] and may be effective in reducing plasma fasting glucose levels and raising high-density lipoprotein cholesterol in people with type 2 diabetes [24]. 

Many of the comorbidities associated with magnesium deficiency are known to occur more frequently among African Americans. For example, diabetes is up to three times more common in people of African or Afro-Caribbean origin [25]. Ethnic differences also exist with hypertension [26], osteoporosis and fractures [27], and frailty [28], with African Americans often at the greatest risk. Given that we observed the lowest magnesium intake in this group, in line with previous research [15,16], supplementation may be of particular importance to this population group. On a societal level, addressing social inequalities in health is a public health priority [29], and magnesium supplementation in groups at risk of deficiency could help to reduce established inequalities in health. Indeed, health inequalities are prevalent in older adults [30] and ethnic disparities in U.S. older adults also exist [31]. 

Findings from the present study add to the wider literature on ethnic differences in dietary intake. Several studies have documented poorer dietary quality among African Americans and better dietary quality among Hispanics [17,18,32]. Potential reasons for ethnic differences in magnesium intake include socioeconomic disparity and cultural differences [33,34,35]. In the U.S., ethnic minorities are more likely, on average, than Caucasians to be from a lower socioeconomic status [36]. Low socioeconomic status has been shown to be strongly associated with poorer overall diet quality, and the disparity in the U.S. continues to widen [32]. The food environment likely plays a role, with affordable healthy foods less accessible but unhealthy convenience foods readily available in more deprived communities [37]. For example, fast food restaurants are geographically associated with predominately black and low-income neighborhoods in the U.S. [38], and a non-white ethnicity is associated with increased fast food consumption [39]. Although it remained significant, the difference in magnesium intake between African Americans and Caucasians was attenuated when indices of socioeconomic status (education and household income) were included in the model, indicating that differences in socioeconomic status partly mediated this association. Interestingly, we observed no significant difference in magnesium intake between Hispanics and Caucasians in the unadjusted model, but when socioeconomic factors were accounted for (i.e., white privilege was adjusted out), mean intake was higher among Hispanics. These findings suggest that differences between Hispanic and Caucasian older adults may be driven by differing dietary traditions and culture. Such dietary traditions and culture will likely be more difficult to alter in older adults and thus intervening in young U.S. ethnic minorities to ensure adequate magnesium intake in later life may be an appropriate strategy.

Strengths of the present study include the large representative sample of U.S. older adults, assessment of magnesium intake from the average of two days’ consumption and adjustment for a range of potential confounders. However, the findings must be interpreted in light of the study’s limitations. First, the dietary assessment relied on participants’ recall of their food intake over 24 h, which, particularly given the older age of the sample, introduced scope for recall bias. Data on magnesium supplementation was not available. Future studies may wish to collect such data for analyses. Data were cross-sectional, so we were unable to examine the extent to which any age-related decline in magnesium intake [15] differs between ethnic groups.

In conclusion, the present study has shown that magnesium intake is low among older adults residing in the U.S., and those of African American ethnicity are at greatest risk of low intake. Targeted interventions to increase magnesium intake in older U.S. adults with a focus on African Americans could help to reduce the burden of morbidity and ethnic inequalities in health in later life.

## Figures and Tables

**Table 1:** Sample characteristics in relation to ethnicity ^a,b^.

	Caucasian	Africa American	Hispanic	Other	*p*
	(*n* = 3438)	(*n* = 1002)	(*n* = 965)	(*n* = 277)	
Age (years), mean (SE)	73.1 (0.1)	72.3 (0.2)	71.5 (0.2)	71.3 (0.4)	<0.001
Sex					0.13
Men	44.1	40.6	42.3	47.2	
Women	55.9	59.4	57.7	252.7	
Weight status					<0.001
Normal	25.6	21.8	18.3	45.3	
Overweight	38.2	31.4	41.1	30.9	
Obesity	36.2	46.8	40.6	23.8	
Education level					<0.001
Below high school	17.1	38.5	56.6	21.2	
High school	26.4	24.2	15.9	22.0	
Higher than high school	56.5	37.3	27.5	56.8	
Annual household income					<0.001
<$25,000	25.6	44.5	50.5	30.2	
$25,000 < $75,000	50.5	45.6	38.5	43.8	
≥$75,000	23.9	11.9	11.0	26.0	
Leisure-time physical activity					<0.001
Inactive	54.6	61.4	66.0	48.7	
Active	45.4	38.6	34.0	51.3	
Smoking status					<0.001
Never	47.6	47.6	53.6	52.9	
Former	45.4	38.7	35.5	35.0	
Current	7.0	13.7	10.9	12.1	
Chronic conditions ^c^					0.419
No	25.1	24.1	28.5	25.7	
Yes	74.9	75.9	71.5	74.3	
Meeting recommended level of dietary magnesium intake					<0.001
No	82.8	90.6	84.4	78.1	
Yes	17.2	9.4	15.6	21.9	
Dietary magnesium intake (mg, day), mean (SE)	276.4 (2.6)	233.0 (4.4)	260.8 (4.1)	285.7 (8.9)	0.028
Total energy intake (kcal, day), mean (SE)	1807.9 (12.9)	1608.4 (25.9)	1634.0 (25.6)	1711.2 (44.5)	<0.001

^a^ Values are percentages unless stated otherwise. ^b^ All estimated are weighted to be nationally representative. ^c^ Chronic conditions includes diabetes, cardiovascular disease, arthritis, and cancer.

**Table 2:** Linear regression models of the association between ethnicity and energy intake adjusted magnesium intake ^a^.

	Beta-Coefficient (95% CI), *p*-Value ^a^					
	Univariate		Age-Adjusted		Fully-Adjusted ^b^	
**Ethnicity**						
Caucasian	Reference		Reference		Reference	
African American	−19.9 (−26.1 to −13.7)	<0.001	−20.6 (−27.0 to −14.3)	<0.001	−13.0 (−18.8 to −7.2)	<0.001
Hispanic	4.9 (−0.9 to 10.8)	0.098	3.6 (−2.4 to 9.6)	0.237	14.0 (7.5 to 20.5)	<0.001
Other	20.7 (6.7 to 34.8)	0.004	19.3 (5.3 to 33.4)	0.008	17.2 (3.8 to 30.5)	0.012

^a^ All models are adjusted for total energy intake. ^b^ Adjusted for age, sex, body mass index, education level, household income, physical activity, smoking status, and chronic conditions.
